# Comparative Study of Microwave and Ultrasound‐Assisted Extraction of Phosvitin From Egg Yolk

**DOI:** 10.1002/fsn3.71576

**Published:** 2026-03-07

**Authors:** Niloofar Zahed, Ali Motamedzadegan, Reza Esmaeilzadeh Kenari, Jafar Mohammodzadeh Milani, Ali Rashidinejad, Anna Lante

**Affiliations:** ^1^ Department of Food Science and Technology Sari Agricultural Sciences and Natural Resources University Sari Iran; ^2^ Riddet Institute, Massey University Palmerston North New Zealand; ^3^ Department of Agronomy, Food, Natural Resources, Animals, and Environment Padova University Padova Italy

**Keywords:** egg yolk, microwave, phosphorus, phosvitin, protein, ultrasound

## Abstract

This study investigated the efficacy of microwave and ultrasound technologies in extracting phosvitin from egg yolk. Extractions were conducted for 4 and 8 min using ultrasound powers of 200, 300, and 400 W, and microwave powers of 180, 360, and 600 W. The %yield, %phosphorus, and %nitrogen of the extracted phosvitin were measured. FT‐IR and SDS‐PAGE analyses were performed to characterize the phosvitin. The superior samples from each extraction method were further evaluated for antioxidant properties, emulsifying activity index (EAI), and emulsion stability index (ESI). Samples treated with microwave power of 180 W for 4 min (MT4P180, 4.68%) and ultrasound power of 200 W for 4 min (UT4P200, 3.77%) had the highest %yield compared to other treatments with a significant difference (*p* < 0.05). The highest ratio of phosvitin to total protein with a significant difference (*p* < 0.05) was observed in microwave samples treated at 600 W for 8 min (MT8P600, 76.68%) and in ultrasound samples treated at 400 W for 8 min (UT8P400, 54.41%). The presence and purity of phosvitin were confirmed by FT‐IR (Peaks at 976 and 1079 correspond to PO_4_^3−^) and SDS‐PAGE (bands in the 31–47 kDa range), respectively. The antioxidant activity of MT8P600 (36.20%) was significantly higher than that of UT8P400 (23.58%) (*p* < 0.05). EAI and ESI in the UT8P400 (24.30–98.5) were significantly higher than those of MT8P600 (20.8–83.7) (*p* < 0.05). Therefore, microwave technology is considered a modern and efficient method for producing phosvitin, a natural and biodegradable antioxidant and emulsifier, for use in food products.

## Graphical abstract

Microwave‐assisted extraction produced phosvitin with greater purity and recovery compared to ultrasound‐based methods. An increase in microwave power and processing time was positively correlated with both phosvitin purity and HDL removal efficiency, as confirmed by FT‐IR analysis and SDS‐PAGE electrophoresis profiles. Additionally, the antioxidant activity of phosvitin extracted using microwave methods was significantly higher than that of phosvitin extracted using ultrasound methods.

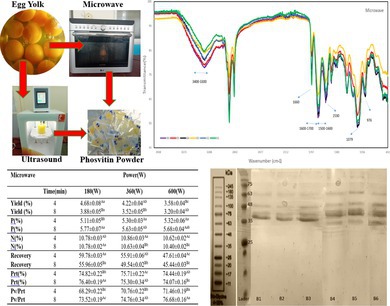

## 1 Introduction

Due to their natural origin, nontoxicity, affordability, and functional versatility—including emulsifying, foaming, gelling, and antioxidant activities—proteins are widely used in food systems (Méx 2008; Hosseini‐Parvar et al. 2009; Alrosan et al. 2025).

Eggs are an essential part of the human diet and are consumed globally (Mine 2002). Despite being composed of about 74% water, eggs are a rich source of high‐quality protein and provide important nutrients such as unsaturated fatty acids, iron, phosphorus, trace minerals, and vitamins A, E, K, and B (Stadelman and Cotterill 1995).

Increased demand for low‐fat, high‐protein egg whites has created an oversupply of yolks, which limits the economic potential of the egg industry (Razi et al. 2023; Nielsen et al. 2024). Developing new products through protein separation and purification based on functional and nutritional properties can enhance the value and use of egg yolk (Huang and Ahn 2019). Egg yolk is composed of two parts: plasma (78%) and granules (2%). Plasma consists of low‐density lipoprotein (85%) and globular glycoprotein (15%). Granules consist of high‐density lipoprotein (73%), low‐density lipoprotein (9%), and 18% phosvitin (Marcet et al. 2022).

Phosvitin constitutes about 11% of egg yolk protein and is a major phosphoprotein of the yolk, with 50%–57% of its amino acids being serine, 90% of which are phosphorylated (Jiang et al. 2019; Yilmaz and Ağagündüz 2020). Phosvitin has an amphiphilic structure with a hydrophilic middle part and a hydrophobic end part. Its high phosphorylation level imparts various physiological activities, such as thermal stability, metal binding, antioxidant activity, and emulsifying properties, making it a promising functional compound (Jiang et al. 2019). As a value‐added protein, phosvitin has potential applications in anti‐inflammatory, antibacterial, antioxidant, and gut health improvement (Thirumalai et al. 2019).

To obtain high‐purity phosvitin, it is essential to remove LDL and HDL. LDL can be easily separated by centrifugation after yolk dilution, but the presence of phosphocalcic bridges between phosvitin and HDL complicates separation. This bridge is formed between the phosphate groups of the remaining phosphoseryl phosvitin and HDL with calcium, which forms the insoluble granule (Zhang et al. 2011). High‐purity phosvitin can be prepared using chromatography (Ren and Wu 2014), but this technique has limitations in practical production due to low throughput and high costs (Zhang et al. 2024).

Various methods have been employed to extract high‐purity phosvitin; however, many have disadvantages, including the presence of residual organic solvents, complicated operations, time consumption, low yield, structural changes, and reduced functional properties of phosvitin (Zhang et al. 2011; Jiang et al. 2019). There is a need for new extraction methods to produce high‐purity phosvitin. Current methods that improve protein extraction efficiency include ultrasound, microwave, enzymatic, pulsed electric field, and supercritical fluid extraction (Görgüç et al. 2019). These eco‐friendly methods offer advantages such as time‐saving, high extraction efficiency, the absence of organic solvent residue, and energy savings (Garcia et al. 2015).

Microwaves have diverse and beneficial effects on proteins, including changes in hydrophilicity and hydrophobicity, structural unfolding, digestibility, denaturation, emulsification, foaming, gel strength, oxidation, and desensitization, depending on power, application time, protein nature, and food type (Deng et al. 2022). Compared to conventional heating, microwaves can accelerate protein unfolding. High‐power microwave heating breaks disulfide bonds, exposing the hydrophobic core to the solvent and depolymerizing the protein, which leads to increased yield and purity (Li et al. 2016; Cao et al. 2019). Microwaves weaken intramolecular and intermolecular forces, including hydrogen bonding, disulfide bonding, and hydrophobic interactions, forming new structures by rearranging molecular forces (Zhu et al. 2018). However, some researchers argue that microwave irradiation does not significantly alter secondary protein structures, primarily affecting tertiary structures (Broz et al. 2024).

Ultrasound extraction accelerates mass transfer and releases proteins through cavitation (Chen et al. 2019). The high extraction efficiency of ultrasound‐assisted extraction is mainly due to its mechanical effects, such as microjets and microcurrents (Li et al. 2017). Previous studies have reported higher protein recovery rates with ultrasound compared to conventional processes, resulting in higher yields and increased solubility and protein recovery (Lafarga et al. 2018; Vernes et al. 2019).

Despite the high importance of phosvitin as a protein with valuable functional and nutritional properties, limited studies have been conducted on the extraction of this compound from egg yolk using modern technologies, such as ultrasound and microwave. This research aimed to develop an innovative, safe, and nontoxic solvent‐free method for phosvitin extraction, thereby maintaining quality and efficiency, and enabling the wider use of this compound in the food industry. The innovation of this research lies in the use of green and nontraditional technologies for phosvitin recovery, which can provide a healthy and cholesterol‐free alternative in products such as mayonnaise and baked goods. In this regard, the present study, for the first time, comprehensively investigates and compares the extraction efficiency, purity, antioxidant activity, emulsification power, and emulsion stability of phosvitin extracted by ultrasound and microwave methods.

## 2 Materials and Methods

### 2.1 Raw Materials

Fresh eggs were purchased from Clay John Company (Kardkoy, Iran), dialysis bags (D9527: 14kd: 43 mm) were from Sigma‐Aldrich, and other chemicals were from Merck. The supplier of dialysis bags and chemicals in Iran was Tamad Kala Company (Tehran, Iran). All chemicals were of analytical grade with purity 99%. A prop ultrasound device, Topsonics model 400 W‐20KHz (UK), and a microwave device, model MA3882RC (South Korea) were used for extraction.

### 2.2 Extraction of Phosvitin

First, the egg white was separated from the yolk. Next, the egg yolk was diluted with an equal proportion of pure water (1:1 ratio). After stirring in ice water for 30 min, the diluted egg yolk solution was centrifuged at 5488 g for 50 min to obtain the precipitate (temperature 8°C). Then, the precipitate was mixed with 4 times its weight of 10% (w/w) ammonium sulfate solution (pH for the mixture = 5.5‐increasing the solubility of phosvitin) and stirred for 3 h in the mixed water/ice bath with a temperature of 0°C (flocculation of insoluble proteins). Then, the mixture was stirred for 15 min in a water bath at 80°C (to denature lipovitellin (HDL)). Ultrasound prop (frequency 20 kHz) with different powers (200, 300, and 400 W) and microwave with different powers (180, 360, and 600 W) were applied separately for 4 and 8 min. The sample temperature was less than 40°C, which was checked by inserting a thermometer into the sample. The ultrasound was applied in a pulsed manner (10‐s pulse and 3‐s rest). To keep the temperature below 40°C, the microwave radiation was applied for 30 s, followed by a 30‐s rest. Also, during the rest period, the sample container was placed in a container containing cold water until the sample temperature reached 25°C. Subsequently, dialysis was performed in distilled water (temperature 25°C) in 5 steps (each step 4 h). After each dialysis step, the distilled water was replaced. Distilled water was not stirred during the dialysis process. Then, the obtained mixture was centrifuged at 5488 g for 20 min (temperature 8°C). Phosvitin powder was finally prepared by separation and drying by freeze‐drying (Jiang et al. 2020).

### 2.3 Determination of Extraction Yield%

The amount of dry weight of extract from 100 g of dried egg yolk was calculated (Ren and Wu 2015).

$$\text{Extraction yield}=\left(\mathrm{Dry}\;\text{weight of extract}/\mathrm{Dry}\;\text{weight of}\;\mathrm{egg}\;\text{yolk}\right)\times 100$$

### 2.4 Determination of Phosphorus% and Phosvitin%

The method of phosphorus determination by Murphy‐Riley was used to perform this assay. The phosphorus content is determined after the ashing process based on the color formed by the reduction of the phosphomolybdate complex in the presence of ascorbic acid (Cho and Nielsen 2024). The amount of phosvitin was multiplied by 10 through the phosphorus content, and the amount of phosvitin was obtained (Ko et al. 2011).

$$\%P\;\left(\text{Percentage of phosphorus}\right)=\left(\text{weight of phosphorus}\;\left(\mathrm{mg}\right)/\mathrm{Dry}\;\text{weight of extract}\;\left(\mathrm{mg}\right)\right)\times 100$$

%Phosvitin=P×10

### 2.5 Determination of Nitrogen % and Total Protein%

The total nitrogen in the extracted phosvitin sample was determined using the Kjeldahl method (AOAC 925.31), which employed the nitrogen coefficient (AOAC 1990; Jiang et al. 2020). The nitrogen coefficient of phosvitin is 7.69. Dividing the amount of phosvitin by the coefficient of 7.69, the amount of phosvitin nitrogen was obtained. Then, the nitrogen of phosvitin is subtracted from the total nitrogen. The remaining nitrogen is multiplied by 5.72 (egg yolk protein conversion factor) (Mariotti et al. 2008) to obtain the amount of nonphosvitin protein. Finally, the total protein is obtained from the sum of phosvitin protein and nonphosvitin protein.

$$\%N\;\text{Phosvitin}=\left(\text{Phosvitin}/7.69\right)\times 100$$

$$\%\text{Protein nonphosvitin}=\left(N-N\;\text{Phosvitin}\right)\times 5.72\times 100$$

$$\%\text{Total protein}=\left(\text{Phosvitin}+\text{Protein nonphosvitin}\right)\times 100$$

where %N is the percentage of nitrogen.

### 2.6 Phosvitin Ratio to Total Protein (% Phosvitin Purity)

The purity of phosvitin was obtained by dividing the percentage of phosvitin % by the total protein (Jung et al. 2013).

$$\%\text{Phosvitin purity}=\left(\text{Phosvitin}/\text{Total protein}\right)\times 100$$

### 2.7 Phosvitin Recovery

Phosvitin contains about 10% phosphorus, and the percentage of phosvitin in the sample was obtained by multiplying the percentage of phosphorus by 10. Then, the percentage of phosvitin was multiplied by the yield. The recovery rate will be calculated assuming 100 g of dried egg yolk contains 4 g of phosvitin (Ko et al. 2011).

$$\%\text{Phosvitin recovery}=\left(\text{Phosvitin}\times \text{Extraction yield}\right)/4$$

The formulas, parameters, and units related to the evaluation of extracted phosvitin are shown in Table 1.

**TABLE 1:** Formulas, parameters, and units related to the evaluation of extracted phosvitin.

Formulas for each test	Unit of parameters
Extraction yield = (Dry weight of extract/Dry weight of egg yolk) × 100	Extraction yield = Expressed as a percentage and has no units Dry weight of extract milligrams Dry weight of egg yolk = milligrams
%P = (weight of phosphorus/Dry weight of extract) × 100 %Phosvitin = *P* × 10	*P* = Expressed as a percentage and has no units. weight of phosphorus = milligrams Dry weight of extract = milligrams Phosvitin = expressed as a percentage and has no units
% N Phosvitin = (Phosvitin/7.69) × 100 %Protein nonphosvitin = (N‐ N Phosvitin) × 5.72 × 100% Total protein = (Phosvitin+ Protein nonphosvitin) × 100	N Phosvitin = Expressed as a percentage and has no units Phosvitin = milligrams Nonphosvitin protein = milligrams
%Phosvitin purity = (Phosvitin/Total protein) × 100	Phosvitin purity = Expressed as a percentage and has no units Phosvitin = milligrams Total protein = milligrams
%Phosvitin recovery = (Phosvitin × Extraction yield)/4	Phosvitin recovery = Expressed as a percentage and has no units Extraction yield = Expressed as a percentage and has no units Phosvitin = Expressed as a percentage and has no units

### 2.8 FT‐IR

0.1 g of phosvitin was placed on a 1 cm diameter disk to measure and identify the protein composition. The measurement of phosvitin composition was investigated from 650 to 4000 cm^−1^ (Ji et al. 2018).

### 2.9 SDS‐PAGE (Sodium Dodecyl Sulfate‐Polyacrylamide Gel Electrophoresis)

SDS‐PAGE was conducted using a 10% polyacrylamide gel, prepared with a 30% acrylamide solution, 1.5 M Tris–HCl (pH 8.8), 0.5 M Tris–HCl (pH 6.8), 10% ammonium persulfate, and TEMED. The proteins in the gel were stained with Coomassie Brilliant Blue, which contained 0.1 M aluminum nitrate, followed by staining in a 10% acetic acid solution. Imaging was then performed. The relative composition of phosvitin in the protein solution was estimated based on band intensity concerning molecular weight (Jung et al. 2013).

In this step, each of the two extraction methods first selected the sample with the highest phosvitin purity. Then, DPPH radical scavenging tests, ESI, and EAI were performed to compare them.

### 2.10 DPPH Assay

The ability of MT8P600 and UT8P400 samples to inhibit DPPH free radicals was evaluated with minor modifications as described by Ahmadian et al. (2023). 0.1 mL of MT8P600 and UT8P400 samples, each at a concentration of 1 mg/mL in water, were mixed with 10 mL of DPPH methanolic solution (at a concentration of 0.004%). After 1 h of incubation at room temperature (25°C) in the dark, the absorbance of the sample was measured at 517 nm. The DPPH radical inhibition power of MT8P600 and UT8P400 samples was calculated using the following equation.

$$\text{DPPH radical inhibition power}\;\left(\%\right)\;\mathrm{by}\;\text{the samples}=\left(\left(A_{0}\_A\right)/A_{0}\right)\times 100$$

where *A*_0_ is the absorption of DPPH radicals, and *A* is the absorption of the extract and radicals.

### 2.11 Emulsifying Activity Index (EAI) and Emulsion Stability Index (ESI)

EAI and ESI were evaluated with slight modifications according to the method of Chen et al. (2013). Sunflower oil (2 mL) was mixed with 18 mL of 10 mg/mL phosvitin solution (UT8P400 and MT8P600). The mixtures were homogenized at 6000 g for 6 min. 10 μL of the emulsion prepared from phosvitin was added to 8 mL of a 0.1% (w/w) SDS solution. The absorbance at 500 nm of the mixture with 0.1% SDS solution as a blank was recorded. EAI was calculated based on the formula:

$$\mathrm{EAI}=2T\times \left(A_{0}\times N\right)/(C\times \left(1-Ø\right)\times 10,000$$

*A*_0_ represents the absorbance at 500 nm, while *N* is the dilution factor. The constant *T* is equal to 2.303, and *C* denotes the concentration of the samples, which is 10 mg/mL. Additionally, ∅ indicates the volume fraction of sunflower oil, set at 10%.

After 30 min, we recorded the absorbance at λ = 500 nm for the emulsions from UT8P400 and MT8P600. The following formula was used to calculate the ESI for the samples from UT8P400 and MT8P600.

$$\mathrm{ESI}=2T\times \left(A_{t}/A_{0}\right)\times 100$$

Here, *A*_*t*_ and *A*_0_ are the absorbance at 30 min and time 0, respectively.

### 2.12 Statistical Analysis

All statistical methods in this study were analyzed using SPSS version 21 software using one‐way analysis of variance (ANOVA), and Duncan's test was used to show the least significant difference at a confidence level higher than 95% (*p*_Value_ ≤ 0.05). The results were shown in the form of mean with standard deviation, and to reduce the error, all experiments were performed in three repetitions. The Excel version 2013 software was used to draw graphs.

## 3 Results and Discussion

### 3.1 Extraction Yield%

The results demonstrated significant differences in phosvitin extraction yield using ultrasound under various conditions of power and time (*p* < 0.05) because ultrasonic power is linked to cavitation intensity, with higher power causing greater cavitation, leading to protein aggregation and precipitation (Jiang et al. 2020). For microwave‐assisted extraction, the phosvitin yield decreased with increasing power at both times (4 and 8 min). At different power levels (180, 360, and 600 W), the yield decreased with longer extraction times. The highest and lowest yields were found in samples with microwave power of 180 W for 4 min (MT4P180, 4.68%) and MT8P600 (3.20%), respectively (Table 2). Researchers noted that the microwave power of 180 W resulted in the highest protein yield. Conversely, at higher power (240 W), longer extraction times led to decreased yields (Lv et al. 2022), which aligns with the current study's findings. Ren and Wu (2015) reported a phosvitin extraction yield of 2.35% using heat, indicating that ultrasound and microwave methods effectively increase phosvitin yield.

**TABLE 2:** Yield, percentage of phosphorus, percentage of nitrogen, percentage of protein, and ratio of phosvitin to protein extracted by microwave under different conditions.

Microwave	Time (min)	Power (W)		
		180 (W)	360 (W)	600 (W)
Yield (%)	4	4.68 ± 0.08^Aa^	4.22 ± 0.04^Ab^	3.58 ± 0.04^Bc^
Yield (%)	8	3.88 ± 0.05^Ba^	3.52 ± 0.05^Bb^	3.20 ± 0.04^Ab^
P (%)	4	5.11 ± 0.05^Bb^	5.30 ± 0.03^Aa^	5.32 ± 0.06^Aa^
P (%)	8	5.77 ± 0.07^Aa^	5.63 ± 0.05^Ab^	5.68 ± 0.04^Aab^
N (%)	4	10.78 ± 0.03^Ab^	10.86 ± 0.03^Aa^	10.62 ± 0.02^Ac^
N (%)	8	10.78 ± 0.02^Aa^	10.63 ± 0.04^Bb^	10.40 ± 0.02^Bc^
Recovery	4	59.78 ± 0.03^Aa^	55.91 ± 0.06^Ab^	47.61 ± 0.04^Ac^
Recovery	8	55.96 ± 0.05^Ba^	49.54 ± 0.02^Bb^	45.44 ± 0.03^Bc^
Prt (%)	4	74.82 ± 0.22^Bb^	75.71 ± 0.22^Ac^	74.44 ± 0.19^Ab^
Prt (%)	8	76.40 ± 0.19^Aa^	75.30 ± 0.34^Ab^	74.07 ± 0.16^Bc^
Pv/Prt	4	68.29 ± 0.22^Bc^	70.76 ± 0.22^Bb^	71.46 ± 0.19^Ba^
Pv/Prt	8	73.52 ± 0.19^Ac^	74.76 ± 0.34^Ab^	76.68 ± 0.16^Aa^

*Note:* Mean with standard deviation. Different lowercase letters indicate significant differences in data in each row. Different uppercase letters indicate significant differences in data in each column (*p* < 0.05, *n* = 3).

Abbreviations: MT4P180, microwave with 4 min power 180 W; MT4P360, microwave with 4 min power 360 W; MT4P600, microwave with 4 min power 600 W; MT8P180, microwave with 8 min power 180 W; MT8P360, microwave with 8 min power 360 W; MT8P600, microwave with 8 min power 600 W; N, nitrogen; P, phosphorus; Prt, protein; Pv/Prt, ratio of phosvitin to protein.

### 3.2 Determination of Phosphorus%

The results indicated that the percentage of phosphorus increased with higher ultrasound power over 4 and 8 min. Phosphorus content rose with longer ultrasound. Durations across all power levels. Sample UT8P400 (4.20%) had the highest phosphorus percentage, while ultrasound power of 200 W for 8 min (UT8P200, 3.78%) had the lowest among the extracted phosvitin samples. Increased power and ultrasound time enhanced cavitation intensity and the number of cavitation pockets, leading to higher phosphorus extraction (Jiang et al. 2020). Similarly, increasing microwave power and time increased the phosphorus levels in the samples. The percentage of phosphorus increased with higher microwave power over 4 min. Samples microwave power of 180 W for 8 min (MT8P180) and MT4P180 had the highest (5.77%) and lowest (5.11%) phosphorus percentages, respectively, among the extracted phosvitin samples. Le et al. (2021), reported phosphorus amounts of 39.5 and 28.73 mg using liquid chromatography and gel filtration chromatography, respectively, which aligns with the findings of this study.

### 3.3 Nitrogen % and Total Protein%

The nitrogen content of the sample reflects the protein percentage in the extracted sample, with phosvitin being the predominant protein. The results indicated that the nitrogen percentage increased with higher power for over 8 min. The highest and lowest protein percentages were observed in UT8P400 (77.18%) and UT8P200 (70.97%), respectively. The ultrasound power of 300 W for 4 min (UT4P300) and the ultrasound power of 300 W for 8 min (UT8P300) samples showed no significant difference in nitrogen percentage (*p* < 0.05). However, this did not apply to the protein amount. Due to the type of dominant protein and its nitrogen content. As shown in Table 3, the UT8P300 sample had more phosphorus than the UT4P300 sample, indicating a higher phosvitin content, which affected the protein percentage. This is because the conversion factor for phosvitin (7.69) (Jung et al. 2013) differs from that of egg yolk protein (5.72) (Mariotti et al. 2008). Because 80% of phosvitin is composed of the amino acid serine (Jiang et al. 2019), which has a direct impact on the protein conversion factor. The results also showed that increasing microwave power for 8 min led to a rise in nitrogen content. The highest and lowest protein‐containing samples were MT8P600 and MT8P180, respectively, consistent with Lv et al. (2022). Researchers found that increasing time and temperature resulted in higher protein extraction (Taheri et al. 2011; Hamzeh et al. 2018). Kendler et al. (2023) also reported that increasing ultrasound time and microwave power to 350 W enhanced protein extraction.

**TABLE 3:** Sample names based on the type of device used in extraction, and the time and power of the device.

Sample	Extraction	Time (min)	Power (W)
MT4P180	Microwave	4	180
MT4P360	Microwave	4	360
MT4P600	Microwave	4	600
MT8P180	Microwave	8	180
MT8P360	Microwave	8	360
MT8P600	Microwave	8	600
UT4P200	Ultrasound	4	200
UT4P300	Ultrasound	4	300
UT4P400	Ultrasound	4	400
UT8P200	Ultrasound	8	200
UT8P300	Ultrasound	8	300
UT8P400	Ultrasound	8	400

### 3.4 Recovery of Phosvitin

Phosvitin recovery is the percentage of phosvitin obtained from the total phosvitin of egg yolk. The results showed (Table 4) that the lowest and highest protein percentages were for UT8P400 (31.39%) and UT4P200 (35.81%) (*p* < 0.05). The results showed (Figure 4) that with increasing microwave power and time, recovery decreased, unlike phosvitin purity. The lowest and highest recovery rates was for samples (MT8P600) (45.44%) and (MT8P180) (59.78%). Marcet et al. (2023) reported a recovery rate of phosvitin ranging from 15% to 62.5%. They explained that the extraction of phosvitin was affected by pH and salt. Le et al. (2021) reported a phosvitin recovery of 30 to 32% in the extraction and purification of phosvitin by liquid chromatography, which was consistent with phosvitin extraction with ultrasound but was lower than phosvitin extraction with microwave.

**TABLE 4:** Yield, percentage of phosphorus, percentage of nitrogen, percentage of protein, and ratio of phosvitin to protein extracted by ultrasound under different conditions.

Ultrasound	Time (min)	Power (W)		
		200	300	400
Yield (%)	4	3.77 ± 0.04^Aa^	3.48 ± 0.05^Ab^	3.52 ± 0.07^Ab^
Yield (%)	8	3.21 ± 0.03^Bb^	3.27 ± 0.02^Ba^	2.99 ± 0.03^Bc^
P (%)	4	3.80 ± 0.05^Ab^	3.81 ± 0.6^Bb^	3.93 ± 0.04^Ba^
P (%)	8	3.78 ± 0.09^Ab^	4.12 ± 0.10^Aa^	4.20 ± 0.12^Aa^
N (%)	4	11.23 ± 0.03^Ab^	11.38 ± 0.02^Aa^	11.13 ± 0.02^Bc^
N (%)	8	10.71 ± 0.02^Bc^	11.39 ± 0.04^Ab^	11.61 ± 0.02^Aa^
Recovery	4	35.81 ± 0.02^Aa^	33.14 ± 0.03^Bc^	34.58 ± 0.02^Ab^
Recovery	8	30.33 ± 0.02^Bc^	33.68 ± 0.03^Aa^	31.39 ± 0.05^Bb^
Prt (%)	4	73.97 ± 0.26^Ab^	74.93 ± 0.36^Ba^	73.79 ± 0.12^Bb^
Prt (%)	8	70.97 ± 0.15^Bc^	75.80 ± 0.27^Ab^	77.18 ± 0.22^Aa^
Pv/Prt	4	51.37 ± 0.03^Bb^	50.84 ± 0.04^Bc^	53.25 ± 0.03^Ba^
Pv/Prt	8	53.26 ± 0.05^Ac^	54.35 ± 0.02^Ab^	54.41 ± 0.04^Aa^

*Note:* Mean with standard deviation. Different lowercase letters indicate significant differences in data in each row. Different uppercase letters indicate significant differences in data in each column (*p* < 0.05, *n* = 3).

Abbreviations: N, nitrogen; P, phosphorus; Prt, protein; Pv/Prt, ratio of phosvitin to protein; UT4P200, ultrasound with 4 min power 200 W; UT4P300, ultrasound with 4 min power 300 W; UT4P400, ultrasound with 4 min power 400 W; UT8P200, ultrasound with 8 min power 200 W; UT8P300, ultrasound with 8 min power 300 W; UT8P400, ultrasound with 8 min power 400 W.

### 3.5 Phosvitin Ratio to Total Protein (% Phosvitin Purity)

The ratio of phosvitin to total protein indicates the purity of phosvitin. The highest was observed in samples extracted using ultrasound UT8P400, 54.41%, and microwave MT8P600, 76.68%. The results demonstrated that increasing power and time in the extraction process led to higher phosvitin purity, as these conditions facilitated the separation and sedimentation of HDL. Lv et al. (2022) found that increasing microwave time and power resulted in Ovomucoid with higher purity (95%) from egg white, highlighting the positive impact of microwaves on protein. Extraction and purity. Researchers also noted that microwave and ultrasound extraction methods enhanced the purity of collagen from skin and bone compared to extraction without pretreatment (Kendler et al. 2023). In a study by Le et al. (2021) on phosvitin extraction using liquid chromatography and gel filtration chromatography, the phosvitin to total protein ratios were reported as 74% and 81%, respectively, which aligns with and confirms the findings of the current study. Researchers observed that applying high pressure and increasing the process time resulted in an increase in the purity of phosvitin from 27% to 40% (Duffuler et al. 2020). Compared to the current study, they had a lower purity percentage, which indicates the superiority of the ultrasound and microwave process over high pressure in the phosvitin extraction process.

### 3.6 FT‐IR Assay

**FIGURE 1:**
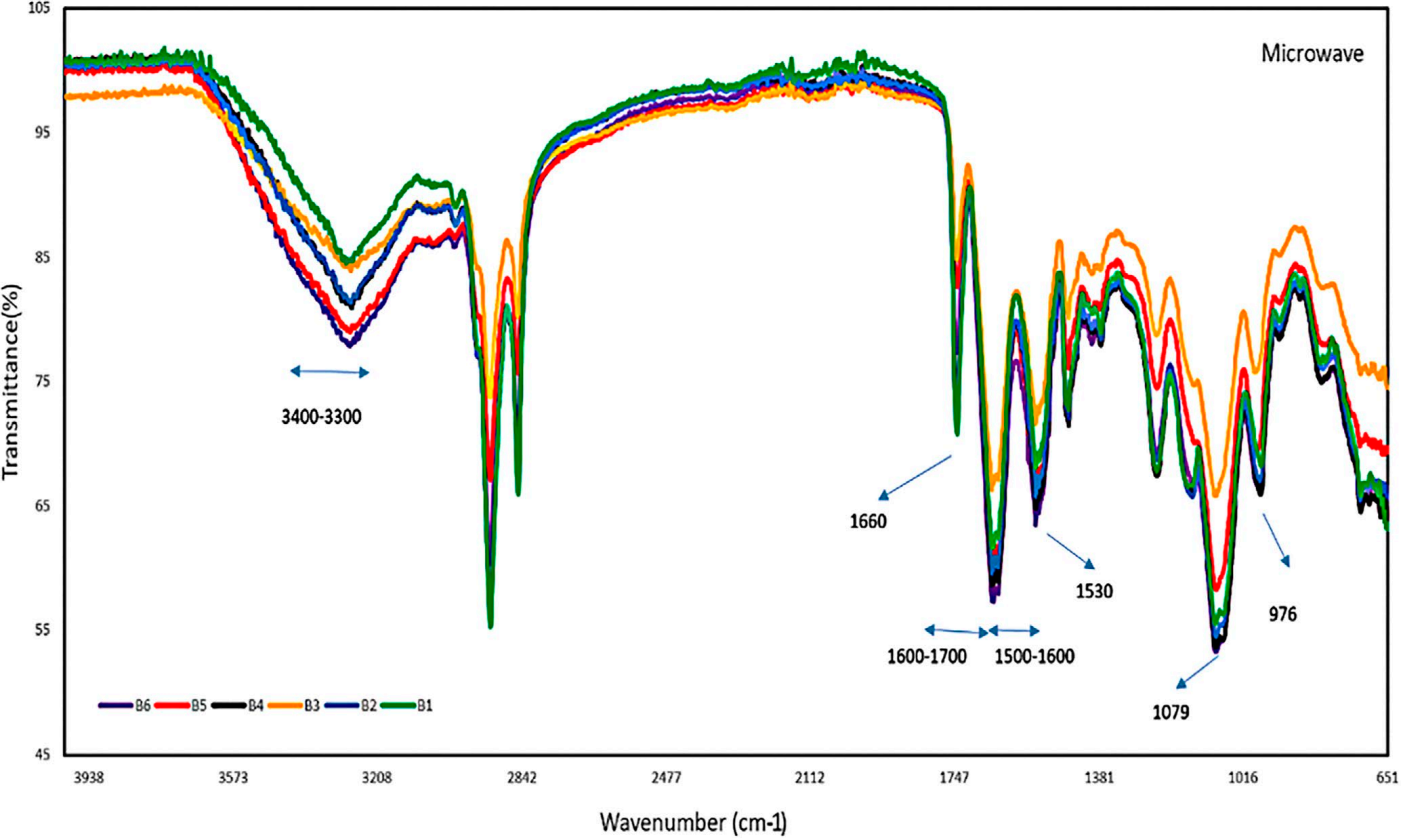
FT‐IR spectrum of a microwave‐extracted phosvitin sample from 650 to 4000 cm^−1^. B1 (microwave with 4 min power 180 W), B2 (microwave with 4 min power 360 W), B3 (microwave with 4 min power 600 W), B4 (microwave with 8 min power 180 W), B5 (microwave with 8 min power 360 W), B6 (microwave with 8 min power 600 W).

**FIGURE 2:**
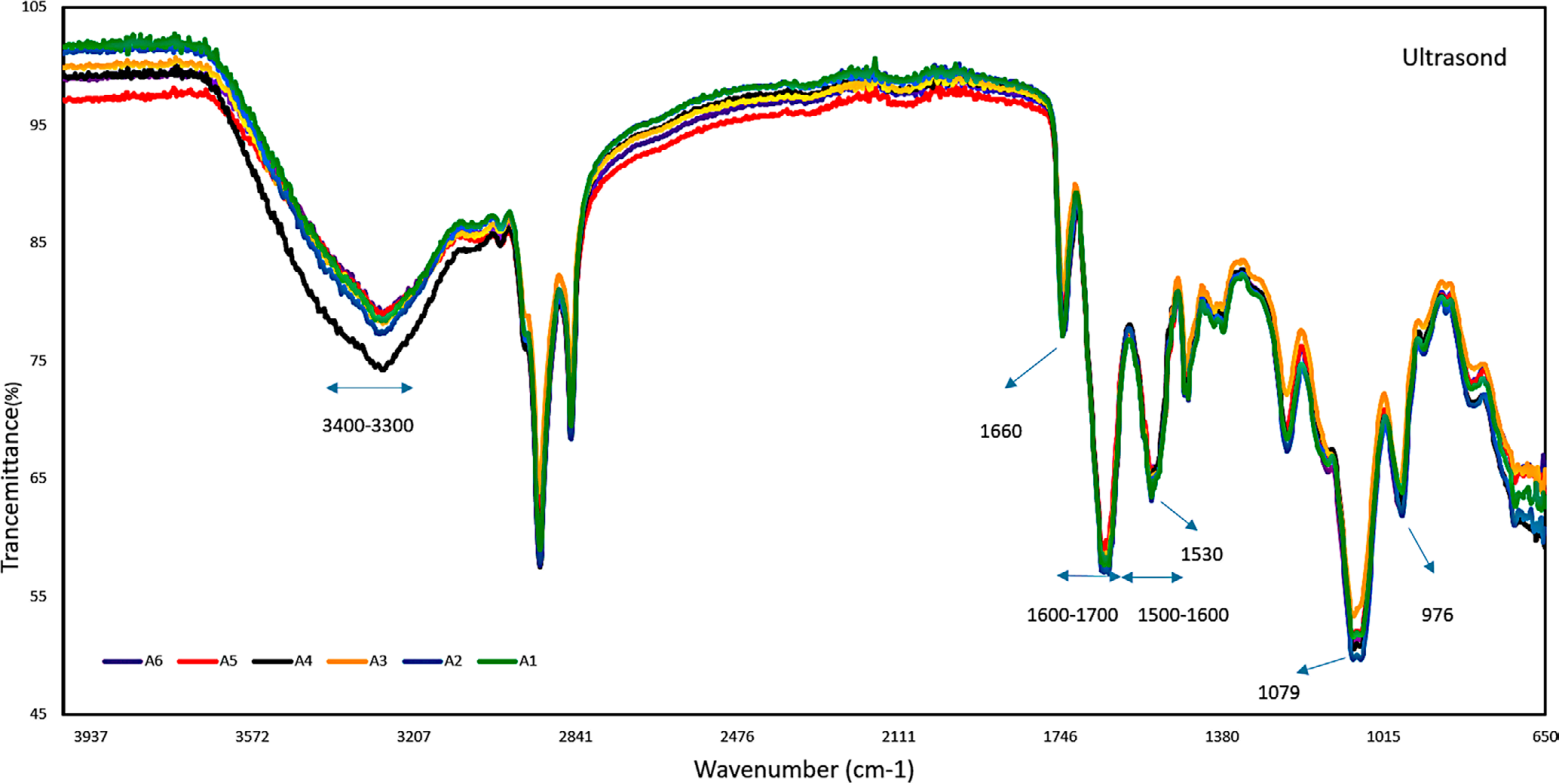
FT‐IR spectrum of ultrasound‐extracted phosvitin sample from 650 to 4000 cm^−1^. A1 (ultrasound with 4 min power 200 W), A2 (ultrasound with 4 min power 300 W), A3 (ultrasound with 4 min power 400 W), A4 (ultrasound with 8 min power 200 W), A5 (ultrasound with 8 min power 300 W), A6 (ultrasound with 8 min power 400 W).

Figure 1 presents the FT‐IR spectrum of phosvitin UE samples, with the peaks of different samples superimposed. This indicates that ultrasound treatment at shorter durations (4 and 8 min) and lower powers (200, 300, and 400 W) has limited purification capability and minimal HDL removal, consistent with Jiang et al. (2020). Figure 2 shows the FT‐IR spectrum for phosvitin ME samples. The peaks at 976 and 1079 correspond to the symmetric and asymmetric stretching vibrations of PO4^3−^, respectively. Additionally, the amide I and II bands at 1600–1700 and 1500–1600, respectively, indicate that phosvitin is a highly phosphorylated protein (Jiang et al. 2019). The bandwidth between 3301 and 3404 is associated with the N‐H stretching vibration of the phosvitin protein. Peaks around 1660 and 1530 indicate C=O stretching in amide I and N–H bending and C–N stretching in amide II (Jiang et al. 2019; Song et al. 2024). The presence of HDL in an ultrasound‐extracted sample resulted in a higher percentage of transmittance in the peak range of 1600–1800, which represents the C=O functional group. On the other hand, the peak of 3200–3400, which represents the O–H functional group with the presence of HDL, has a lower percentage of transmittance (Liu et al. 2002). The change in the percentage of transmittance in the mentioned peaks in both ultrasound and microwave extractions proves the higher presence of HDL in ultrasound‐extracted phosvitin. Researchers have also noted that ultrasound and microwave treatments do not alter the secondary structure of the protein at low power and low temperature (Jiang et al. 2020; Broz et al. 2024), which aligns with and confirms the findings of the current study.

### 3.7 SDS‐PAGE Assay

**FIGURE 3:**
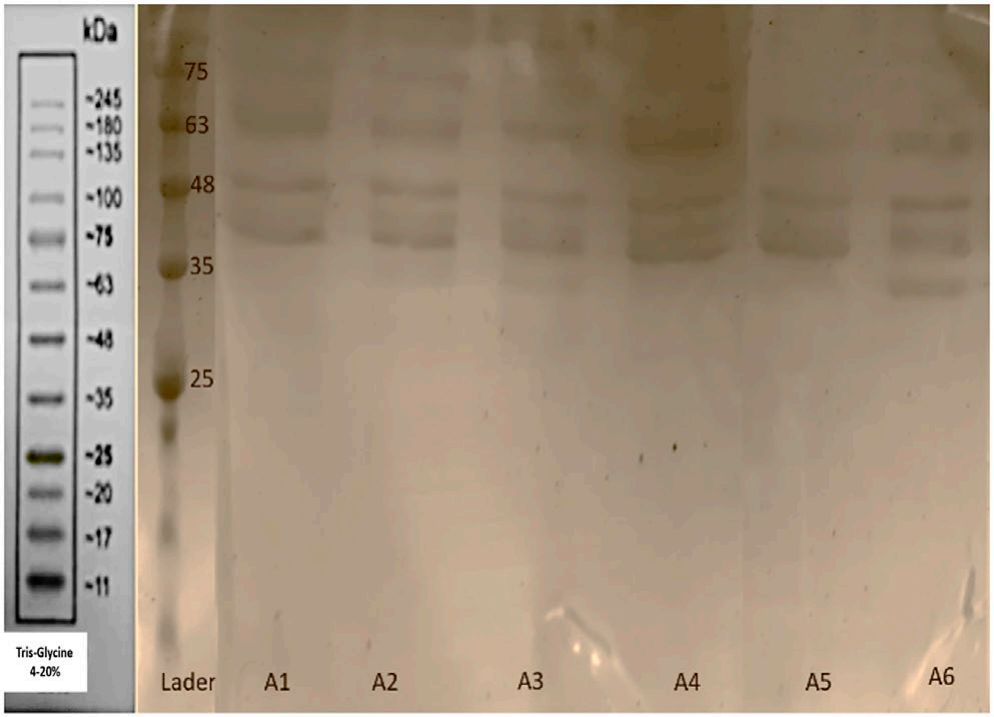
SDS‐PAGE for microwave‐extracted phosvitin. B1 (microwave with 4 min power 180 W), B2 (microwave with 4 min power 360 W), B3 (microwave with 4 min power 600 W), B4 (microwave with 8 min power 180 W), B5 (microwave with 8 min power 360 W), B6 (microwave with 8 min power 600 W).

**FIGURE 4:**
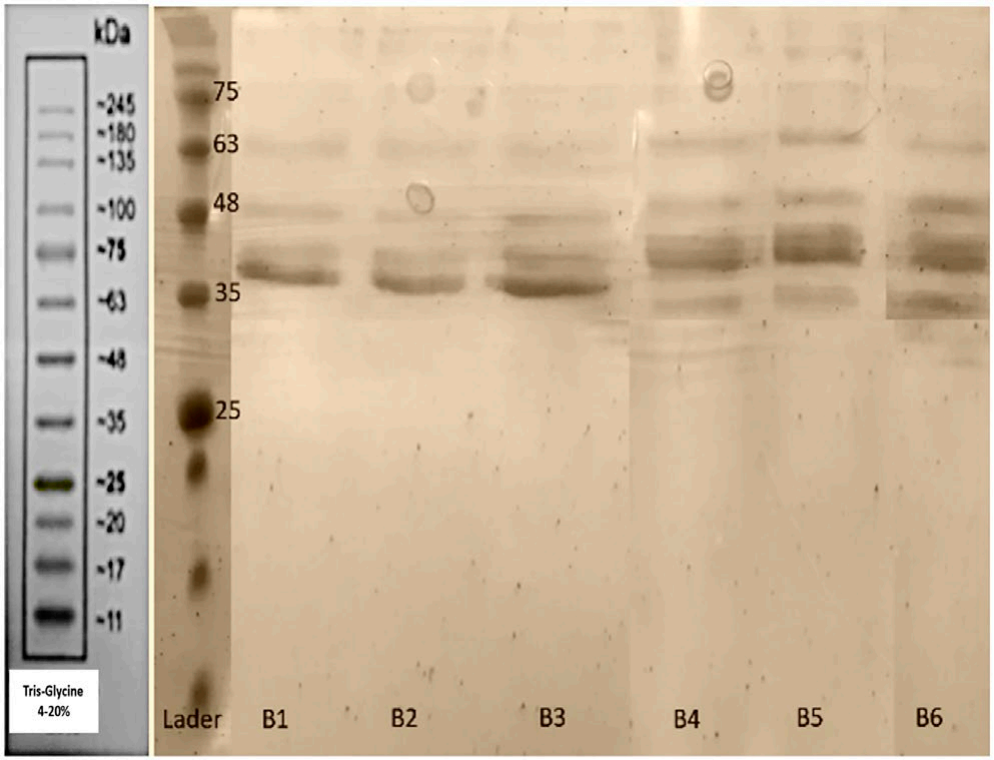
SDS‐PAGE for ultrasound‐extracted phosvitin. A1 (ultrasound with 4 min power 200 W), A2 (ultrasound with 4 min power 300 W), A3 (ultrasound with 4 min power 400 W), A4 (ultrasound with 8 min power 200 W), A5 (ultrasound with 8 min power 300 W), A6 (ultrasound with 8 min power 400 W).

The bands observed in the 45–35 kDa range in Figures 3 and 4 correspond to the phosvitin protein extracted using microwave and ultrasound methods. According to Ren and Wu (2015), bands in the 31–47 kDa range are indicative of phosvitin, confirming that phosvitin exists in different fractions. The presence of phosvitin was confirmed by Duffuler et al. (2020) with a similar molecular weight. Le et al. (2021) also identified phosvitin fractions with molecular weights of 35, 41, and 47 kDa using liquid chromatography, which supports these findings. Conversely, bands at 116, 90, 66, and 15 kDa are associated with HDL protein (Jiang et al. 2020). The results demonstrated that ultrasound‐extracted phosvitin exhibited a variety of bands with different molecular weights, with the UT8P400 sample showing the best protein band. However, HDL protein bands were present in all samples, indicating that ultrasound at 200–400 W for 4–8 min was insufficient for separating HDL and purifying phosvitin. Higher power and longer durations are necessary. In the UT8P400 sample, which had the highest cavitation time and power, phosvitin peaks increased, while HDL bands decreased. Researchers noted that increased ultrasound time led to more cavitation bubbles and higher instantaneous temperatures, causing HDL denaturation. They used 600 W for 10 s for optimal phosvitin purity, cautioning that excessive time and power denature and precipitate phosvitin (Jiang et al. 2020). Microwave extraction for 8 min resulted in more diverse phosvitin bands, with three distinct bands observed, highlighting the effectiveness of this method. At 4 min, HDL bands were more prominent, but the MT8P600 sample showed higher phosvitin purity. Increased microwave time and power led to more uniform electrophoretic bands and HDL denaturation. Microwave extraction enhances intramolecular interactions, particularly electrostatic interactions and hydrogen bonds, while destabilizing the protein. The solvent's hydrogen bond network likely causes HDL contraction. This suggests that protein groups such as –OH, –NH_2_, and –COOH are affected by microwaves, leading to bond‐breaking and fundamental changes in protein structure (Broz et al. 2024).

Samples MT8P600 (microwave) and UT8P400 (ultrasound) were selected for their high phosvitin purity. Subsequent DPPH radical scavenging, ESI, and EAI tests were conducted to compare these samples.

### 3.8 EAI and ESI

The results showed (Table 5) that EAI and ESI in the UT8P400 sample were higher (24.30–98.5) than in the MT8P400 sample (20.8–83.7), respectively (*p* < 0.05). This is because the ultrasound sample contains HDL, a phospholipid compound that has better emulsifying properties than phosvitin. The higher of these two parameters indicates the flexibility of the sample, which is essential for emulsion stability (Suhag et al. 2021). In a study, Jiang et al. (2019) reported the value of EAI (27.14) and ESI (103.27) for extracted phosvitin, which is similar to and confirms the results obtained from the current study. Researchers reported the EAI and ESI levels in egg yolk as 49.11 and 90.00, respectively, which had the highest emulsifying properties compared to various milk, wheat, pea, and potato proteins, which they attributed to LDL and HDL (Büyükcan and Karakaya 2021), which were higher in comparison to the studied phosvitin. This is because heat (80°C for 15 min) was applied in the phosvitin extraction process. Proteins such as LDL and HDL are denatured under heat (above 65°C) (Campbell et al. 2005). However, the EAI of phosvitin obtained by microwave in this study was higher than that of wheat protein isolate (11.60). The ESI of phosvitin obtained by microwave in this study was higher than that of wheat protein isolate (79.33), pea (74.33), potato protein isolate (78.67), and equal to that of whey protein concentrate (84.17) and buttermilk powder (82.67), determined by Büyükcan and Karakaya (2021). These findings indicate the high functional value of phosvitin in terms of its emulsifying properties.

**TABLE 5:** Evaluation of EAI and ESI and DPPH free radical scavenging for samples MT8P600 and UT8P400.

Sample	DPPH	EAI	ESI
MT8P600	36.20 ± 1.55^a^	20.8 ± 1.6^b^	83.7 ± 0.47^b^
UT8P400	23.58 ± 2.3^b^	24.30 ± 0.71^a^	98.5 ± 0.98^a^

*Note:* Mean with standard deviation. Different lowercase letters indicate significant differences in data in each column (*p* < 0.05, *n* = 3).

Abbreviations: EAI, emulsifying activity index; ESI, emulsion stability index; MT8P600, microwave at 8 min at power 600 W; UT8P400, ultrasound with 8 min power 400 W.

### 3.9 DPPH Radical Inhibition

The results in Table 2 showed that the antioxidant properties of the MT8P400 sample (36.20%) are higher than those of the UT8P400 sample (23.58%) and have higher radical inhibition (*p* < 0.05). Because the UT8P400 sample has lower purity. A phosphocalcite bridge is established between HDL and phosvitin, which prevents the inhibition of DPPH radicals (Zhang et al. 2024). On the other hand, the MT8P400 sample has a higher percentage of phosvitin than UT8P400, in addition to being more pure. In the study of Jung et al. (2013), the extracted phosvitin antioxidant properties were expressed between 21% and 63%. Additionally, in another study, researchers reported that phosvitin inhibited DPPH radicals by 10% (Jiang et al. 2019). On the other hand, in a study, the phosvitin antioxidant power was reported to be between 27.2% and 53% (Jiang et al. 2020). Song et al. (2024) reported the highest and lowest DPPH radical inhibition values by phosvitin to be 42% and 21%, respectively (Song et al. 2024), which is similar to and confirms the results obtained from the current study.

## 4 Conclusion

In this investigation, we have successfully demonstrated the efficacy of both microwave and ultrasound technologies for phosvitin extraction from egg yolk across various power settings and exposure durations. Microwave‐assisted extraction exhibited superior performance, yielding phosvitin with enhanced purity and greater recovery rates compared to ultrasound‐assisted methodologies. The experimental evidence indicates that increasing both microwave power and processing time positively correlates with phosvitin purity and HDL removal efficiency, as corroborated by FT‐IR spectroscopic analysis and SDS‐PAGE electrophoretic profiles. Notably, ultrasound‐assisted extraction conducted at lower temperatures and shorter durations preserved the native protein conformation, suggesting its utility when protein structural integrity is paramount. However, for applications prioritizing phosvitin purity, extended processing times and elevated power parameters during ultrasound treatment are recommended. The successful isolation of high‐value phosvitin from egg yolk represents a significant advancement toward addressing the current utilization imbalance between egg white and yolk fractions, thereby contributing to the economic and environmental sustainability of the egg processing industry. The implementation of these novel microwave and ultrasound methodologies not only establishes a reliable foundation for subsequent purification protocols but also advances eco‐friendly valorization strategies for high‐value food ingredients. Furthermore, the recovered phosvitin, with its demonstrated emulsifying and antioxidant properties, presents considerable potential for diverse food applications. These include functioning as a natural emulsifier in complex food systems, serving as a protective carrier in nano‐emulsions for environmentally and oxidatively sensitive bioactive compounds, and as a functional ingredient in biodegradable edible films. These applications underscore the multifunctional capacity of phosvitin as a technologically versatile and nutritionally valuable component for next‐generation food formulations.

## Author Contributions

Niloofar Zahed: formal analysis (equal), methodology (equal), software (equal), visualization (equal), writing – original draft. Ali Motamedzadegan: investigation (equal), project administration (equal), resources (equal), supervision (equal), writing – review and editing (equal). Reza Esmaeilzadeh Kenari: conceptualization (equal), data curation (equal), investigation (equal), project administration (equal), supervision (equal), writing – review and editing (equal). Jafar Mohammodzadeh Milani: investigation (equal), project consultation (equal). Ali Rashidinejad: investigation (equal), project consultation (equal). Anna Lante: investigation (equal), project consultation (equal).

## Funding

The authors have nothing to report.

## Conflicts of Interest

The authors declare no conflicts of interest.

## Data Availability

Research data are not shared.
